# Baseline values for Quantra^®^ QPlus^®^ in healthy pregnant women at term and comparison to standard laboratory coagulation values: a prospective observational study

**DOI:** 10.1186/s12884-025-07927-z

**Published:** 2025-08-08

**Authors:** Suthawan Anakmeteeprugsa, Antonio Gonzalez-Fiol, Angelique Garay, Hung-Mo Lin, Zili He, Aymen Alian

**Affiliations:** 1 Department of Anesthesiology, Yale University School of Medicine, PO Box 208051, 333 Cedar Street, New Haven, CT 06520 USA; 2 Yale Center for Analytical Sciences, Yale University School of Public Health, New Haven, CT USA; 3 Faculty of Medicine Siriraj Hospital, Department of Anesthesiology, Golden Jubilee Medical Center , Mahidol University, Nakhon Pathom, Thailand

**Keywords:** Blood coagulation test, Hypofibrinogenemia, Point-of-care viscoelastic testing, Postpartum hemorrhage

## Abstract

**Background:**

Hypofibrinogenemia is associated with progression from moderate to severe postpartum hemorrhage (PPH). Early recognition and replacement of fibrinogen are emphasized during the management of PPH. The Quantra^®^ QPlus^®^ System, a novel point-of-care viscoelastic testing (POCVT) device, has been designed to provide rapid assessment of hemostasis. We aimed to evaluate the correlation between Quantra parameters and standard laboratory coagulation tests, and to establish baseline Quantra values in healthy term pregnant women.

**Methods:**

Healthy pregnant women in labor or scheduled for elective cesarean delivery (CD) were enrolled in our prospective observational study. Blood samples for Quantra and standard laboratory coagulation tests were taken simultaneously. Quantra values, standard laboratory coagulation test, time of blood collected, and time to the result were recorded. We compared the baseline values between CD and labor group using a *t*-test, and the correlation between Quantra and standard laboratory coagulation test was calculated using partial Pearson correlation.

**Results:**

170 healthy pregnant women were included; 126 cases were in the CD group, and 44 patients were in the labor group. We found a strong correlation between Quantra Fibrinogen contribution to Clot Stiffness (FCS) and fibrinogen level (*r* = 0.67). The median [interquartile range] time of fibrinogen results by Quantra was 36 [28, 48] minutes faster than the standard laboratory coagulation tests. Baseline ranges for Quantra values, which were not significantly different between the two groups, demonstrated hyperfibrinogenemia during pregnancy.

**Conclusion:**

Quantra is a novel POCVT device that rapidly provides coagulation status in pregnant women. The strong correlation between FCS and fibrinogen level can be helpful for early recognition of hypofibrinogenemia for the management of PPH.

## Introduction

Postpartum hemorrhage (PPH) remains one of the major causes of maternal morbidity and mortality worldwide, responsible for 25% of maternal deaths [1, 2]. Maternal morbidity from PPH is associated with acute respiratory failure, renal failure, sepsis, blood transfusion-related complications, and prolonged hospitalization [3, 4]. Over a decade of research has highlighted the importance of plasma fibrinogen as an early predictor for severe obstetric hemorrhage. Hypofibrinogenemia, defined as fibrinogen levels lower than 2 g/L, is associated with progression from moderate to severe PPH with a 100% positive predictive value [5–8]. Besides, plasma fibrinogen is rapidly consumed and should be replaced early in the setting of severe PPH. The known long turnaround times of conventional laboratory tests (up to 60 min) [9–11], has favored the use of point of care viscoelastic testing (POCVT). This technology allows for bedside analysis of whole blood, and results can be obatined within 15 min [11–14].

The POCVT devices provide quick results of patient’s coagulation status and aid in the management of complex bleeding cases resulting in reducing blood product transfusions and improving patient outcomes [12, 13]. POCVT devices include thromboelastography (TEG^®^) and rotational thromboelastometry (ROTEM^®^) and recently a novel device called the Quantra System. The Quantra System is a cartridge-based system based on an ultrasound technology called Sonic Estimation of Elasticity via Resonance (SEER) sonorheometry. SEER sonorheometry works by inducing shear wave resonance within a blood sample. The characteristics of the resonance are analyzed to evaluate the shear modulus of elasticity. Contrary to other point of care viscoelastic testing devices, the Quantra System technology allows for analysis of the hemostasis function without disrupting the clot [15, 16]. Previous studies have reported the use of Quantra with the QPlus^®^ or QStat^®^ Cartridge in a variety of perioperative settings that may face severe bleeding (e.g., cardiac, trauma, and orthopedic patients, including pregnant women) [13, 15, 17–19].

Management of obstetric hemorrhage may be improved by establishing reference ranges for the Quantra QPlus System that are specific to the obstetric population, which is known to be in a hypercoagulable state. However, there is currently a lack of standardized data and defined normal values for Quantra QPlus parameters in obstetric care. The aim of this study is to determine the correlations between Quantra parameters and standard laboratory coagulation tests, and the secondary outcome is to establish the baseline values of the Quantra QPlus System in healthy pregnant women at term.

## Materials and methods

### Study population and study protocol

This prospective observational study was approved by the Yale University institutional review board (IRB no.2000031959). Informed consent was obtained from all subjects. The study took place from July 2022 to June 2023. Healthy pregnant women (18 years and older) with uncomplicated pregnancies at full-term gestation (38 to 40 weeks) were enrolled after presenting for elective cesarean delivery (CD) or for labor. The exclusion criteria included pregnant women with HELLP syndrome (hemolysis, elevated liver enzymes and low platelet count); hemoglobin level below 9 g/dL; gestational thrombocytopenia (< 100 × 10^9^/L); bleeding disorders; and pregnant women receiving anticoagulant drugs for thromboembolism or mechanical heart valve.

For laboring women and scheduled CD cases, Quantra^®^ sampling occurred with routine laboratory work at the time of insertion of an intravenous cannula. One ethylenediaminetetraacetic acid (EDTA) tube was collected for the complete blood count (CBC), in accordance with our standard of care. Two 2.7 mL citrated blue-top tubes containing 3.2% sodium citrate were collected: one was sent to the hematology laboratory for standard laboratory coagulation tests, including partial thromboplastin time (PTT), prothrombin time (PT), international normalized ratio (INR), and fibrinogen level; the second sample was used with the Quantra device. The Quantra System was operated using the QPlus Cartridge.

For each enrolled subject, in addition to Quantra and standard laboratory coagulation test results, demographic data (age, race, ethnicity, height, weight, gestational age), time of blood collection, and time to result were recorded. The de-identified data was entered into a password-secured program.

According to the International Federation of Clinical Chemistry (IFCC) and the Clinical Laboratory Standard Institute (CLSI), a reference interval is defined as the interval between and including two reference limits (at the 2.5th percentile and 97.5th percentile) which contains the central 95% of a reference population. The test results from a healthy population are needed to form a normal distribution as a reference population, and 120 samples are enough to provide that interval [20–22].

### Quantra QPlus system

**Fig. 1:**
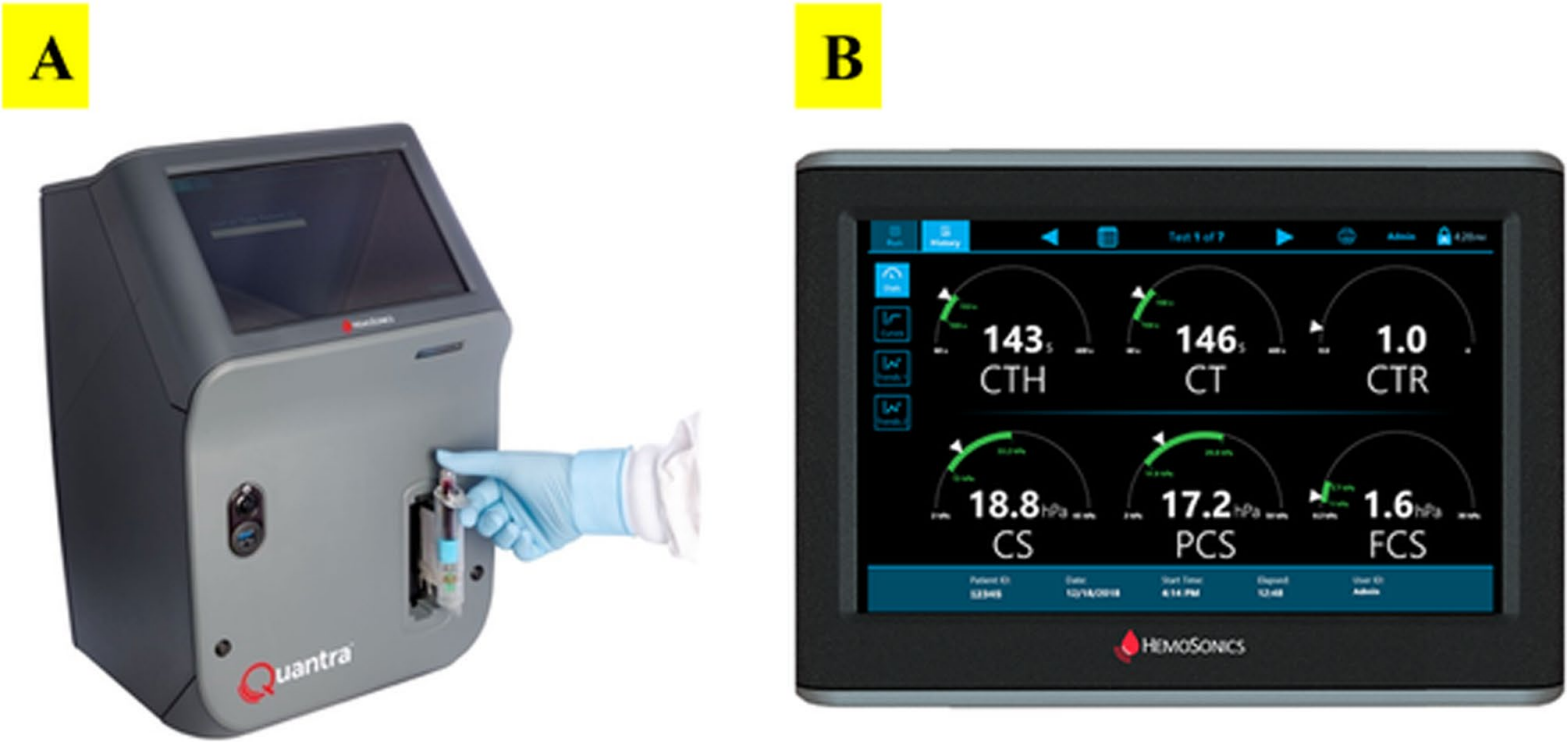
**(A)** Quantra device with insertion of an inverted blood sample (**B)** The screen of the Quantra device displays 6 parameters: Clot Time with Heparinase (CTH), Clot Time (CT), Clot Time Ratio (CTR), Clot Stiffness (CS), Platelet Contribution to clot Stiffness (PCS), Fibrinogen Contribution to clot Stiffness (FCS)

The Quantra QPlus system is a cartridge-based device that uses ultrasound-induced resonance to measure clot time and clot stiffness. Once the whole blood sample was obtained in an evacuated tube, it was gently inverted and then, connected to the Quantra cartridge to start the process of measurement (Fig. 1 A). The QPlus Cartridge output displays 6 parameters which are Clot Time (CT), Clot Time with Heparinase (CTH), Clot Time Ratio (CTR), Clot Stiffness (CS), Fibrinogen Contribution to clot Stiffness (FCS) and Platelet Contribution to clot Stiffness (PCS) as shown in Fig. 1B. There are 2 calculated parameters which are PCS and CTR. The PCS derives from the difference between CS and FCS. The CTR is calculated from the ratio of CT over CTH and provides a measure of the heparin effect with a value more than 1.4 [19].

### Statistical analysis

Descriptive statistics of the baseline Quantra^®^ and standard laboratory coagulation test values were presented as mean ± standard deviation (SD) and median [interquartile range; IQR] for each of the two delivery groups. Normality assumption was evaluated by visual inspection of the histogram and quantile-quantile plot (Q-Q plot). Comparisons between the CD and labor groups were performed using the 2-sample *t*-tests, assuming equal variance, p-value of less than 0.05 was considered statistically significant. Because of their similar distributions, the two groups were subsequently combined. Summary statistics included mean ± SD and median [IQR], and the 2.5th percentile and the 97.5th percentile. Correlations between Quantra^®^ and standard laboratory coagulation tests were estimated using partial Pearson correlation which adjusted for age, body mass index (BMI), race, and ethnicity.

## Results

We enrolled 197 pregnant women. Three patients were excluded due to pre-analytical sample issues (two samples with inadequate blood volumes and one clotted sample), 24 patients were excluded due to incomplete standard laboratory coagulation results. 170 full-term pregnant women (mean gestational age 39 weeks ± 2 days) patients were included in the analysis, with 126 patients in CD group, and 44 patients in labor group. Patient demographic data were summarized in Table 1. There was no significant difference between the two groups in any of the baseline values of the standard laboratory coagulation tests and Quantra device (Table 2). The mean ± SD, median [IQR], the 2.5th percentile and the 97.5th percentile of the results from standard laboratory coagulation tests and the Quantra device in all 170 pregnant women were demonstrated in Table 3.

**Table 1:** Baseline characteristics

	Total (*n* = 170)	CD (*n* = 126)	Labor (*n* = 44)
Age (years)	32.5 ± 5.4	33.9 ± 4.7	28.4 ± 5.4
BMI (kg/m^2^)	33.3 ± 7.2	33.6 ± 7.2	32.6 ± 7.3
Race			
White	107 (62.9%)	79 (62.7%)	28 (63.6%)
Black	29 (17.1%)	21 (16.7%)	8 (18.2%)
Asian	10 (5.9%)	8 (6.3%)	2 (4.6%)
Other	24 (14.1%)	18 (14.3%)	6 (13.6%)
Ethnicity			
Hispanic	32 (18.8%)	19 (15.1%)	13 (29.5%)
Non-Hispanic	138 (81.2%)	107 (84.9%)	31 (70.5%)

The data are presented as mean ± SD or *n* (%)

*Abbreviations*: *BMI* body mass index, *CD* cesarean delivery

**Table 2:** The comparison of baseline values from standard laboratory coagulation tests and Quantra between Cesarean delivery (CD) and labor group

Variables	Mean ± SD		*P*-value	Median [IQR]		*P*-value
	CD (*n* = 126)	Labor (*n* = 44)		CD (*n* = 126)	Labor (*n* = 44)	
Laboratory Values						
Platelets (x10^9^/L)	232.2 ± 54.4	240.9 ± 66.5	0.39	225.0 [196.0-267.0]	225.0 [206.0-285.0]	0.62
Fibrinogen (mg/dL)	482.0 ± 87.2	474.4 ± 70.5	0.61	464.0 [432.0-522.0]	456.5 [422.0-517.5]	0.48
INR	0.91 ± 0.04	0.91 ± 0.03	0.52	0.9 [0.88–0.93]	0.9 [0.89–0.93]	0.32
PT (sec)	9.6 ± 0.4	9.5 ± 0.3	0.29	9.6 [9.3–9.8]	9.5 [9.3–9.8]	0.46
PTT (sec)	25.7 ± 1.8	25.5 ± 1.6	0.62	25.7 [24.3–27]	25.4 [24.9–26.5]	0.76
Quantra^®^ Values						
CT (sec)	131.0 ± 12.3	131.7 ± 11.8	0.75	130.0 [124.0-137.0]	133.0 [126.5–139.0]	0.26
CTH (sec)	125.2 ± 11.1	125.4 ± 11.6	0.92	125.0 [118.0-133.0]	126.5 [119.0-134.0]	0.49
CTR	1.05 ± 0.06	1.05 ± 0.06	0.96	1.0 [1.0-1.1]	1.1 [1.0-1.1]	0.83
CS (hPa)	30.9 ± 7.1	29.5 ± 6.9	0.29	30.9 [26.0-34.8]	27.9 [24.6–32.8]	0.18
PCS (hPa)	26.1 ± 5.6	25.3 ± 5.6	0.38	26.0 [22.5–29.4]	24.5 [21.6–28.6]	0.23
FCS (hPa)	4.7 ± 1.8	4.3 ± 1.5	0.14	4.6 [3.5–5.7]	3.8 [3.3–4.8]	0.11

The *p*-values are based on the 2-sample *t*-tests

*Abbreviations*: *CT* Clot time (seconds-sec), *CTH * Clot Time with Heparinase (seconds-sec), *CTR * Clot Time Ratio = CT/CTH, *CS* Clot Stiffness (hectoPascals - hPa), *FCS *Fibrinogen Contribution to clot Stiffness (hectoPascals - hPa), *INR* International normalized ratio, *IQR* Interquartile range, *PCS* Platelet Contribution to clot Stiffness (hectoPascals - hPa), *PT* Prothrombin time(seconds-sec), *PTT* Partial thromboplastin time(seconds-sec), *SD* Standard deviation

**Table 3:** The baseline values from standard laboratory coagulation tests and Quantra for all patients

**Variables** **(Reference range)**	**Total (n= 170)**			
	**Mean ± SD**	**Median [IQR]**	2.5^th^percentile	97.5^th^percentile
Laboratory Values				
Platelets (150 - 420 x10^9^ /L)	234.4 ± 57.7	225.0 [197.0-270.0]	139	365
Fibrinogen (194 - 448 mg/dL)	480.0 ± 83.1	462.5 [428.0-522.0]	342	679
INR (0.92 – 1.23)	0.9 ± 0.04	0.9 [0.89-0.93]	0.86	0.99
PT (9.6 – 12.3 sec)	9.6 ± 0.4	9.5 [9.3-9.8]	9.0	10.4
PTT (23.0 - 31.4 sec)	25.7 ± 1.8	25.7 [24.5-26.7]	22.0	28.9
Quantra^®^ Values				
CT (104 – 166 sec)	131.2 ± 12.2	130.0 [125.0-138.0]	110	154
CTH (103-153 sec)	125.2 ± 11.2	125.0 [118.0-133.0]	105	146
CTR (< 1.4)	1.05 ± 0.06	1.1 [1.0-1.1]	1.0	1.2
CS (13-33.2 hPa)	30.5 ± 7.1	29.8 [25.7 – 34.7]	18.8	45.8
PCS (11.9-29.8 hPa)	25.9 ± 5.6	25.3 [22.1-29.4]	16.5	37.0
FCS (1.0-3.7 hPa)	4.6 ± 1.7	4.3 [3.4-5.4]	2.3	9.0

*Abbreviations*: *CT* Clot time (seconds-sec), *CTH* Clot Time with Heparinase (seconds-sec); CTR = Clot Time Ratio = CT/CTH, *CS* Clot Stiffness (hectoPascals - hPa), *FCS* Fibrinogen Contribution to clot Stiffness (hectoPascals - hPa), *INR *International normalized ratio, *IQR* Interquartile range, *PCS* Platelet Contribution to clot Stiffness (hectoPascals - hPa), *PT* Prothrombin time(seconds-sec) *PTT *Partial thromboplastin time(seconds-sec), *SD* Standard deviation

The median total time to fibrinogen result from the standard laboratory coagulation tests was 48 (40, 60) minutes, whereas the Quantra provided completed results within 12.3 (12.1, 12.4) minutes after the blood sample was inserted. The median within-patient difference between these two devices was 36 (28, 48) minutes.

**Fig. 2:**
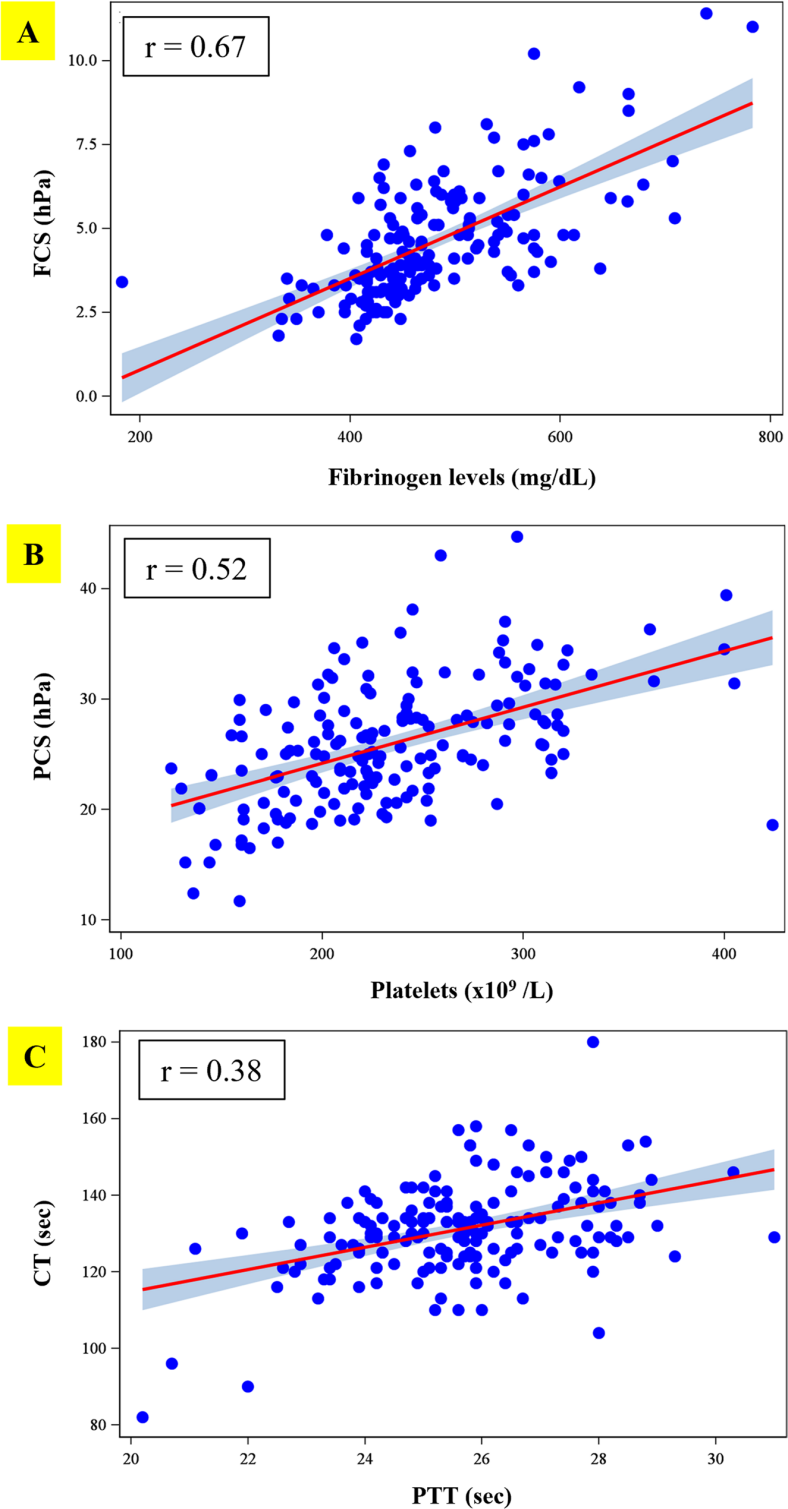
(**A**) The correlation between FCS and fibrinogen level (r=0.67) (**B**) The correlation between PCS and platelet (r=0.52) (**C**) The correlation between CT and PTT (r=0.38). Abbreviations: CT = Clot time; FCS = Fibrinogen Contribution to clot Stiffness; PCS = Platelet Contribution to clot Stiffness; PTT= partial thromboplastin time; sec= seconds; hPa= hectopascal

 By using a partial Pearson correlation adjusted for age, BMI, race and ethnicity, there was a high correlation between FCS and fibrinogen level with r=0.67 (Fig.2A). When comparing PCS to platelet count, a moderate correlation was noted (r=0.52) as shown in Fig.2B. CT showed a weak correlation to PTT (r=0.38) (Fig.2C).

## Discussion

Physiologic change of the hematological system is one of the adaptive mechanisms to minimize postpartum bleeding. Increasing fibrinogen levels, von Willebrand factor, clotting factors (VII, VIII, IX, X, XII) and reduction in fibrinolytic activity lead to a controlled hypercoagulable state [23, 24]. Platelet counts may not change or decrease, while PT and PTT are usually shortened [25–27]. The standard laboratory coagulation values in all 170 participants, as shown in Table 3, were consistent with the hypercoagulability and hyperfibrinogenemia states observed during pregnancy [27, 28]. In our subgroup of patients, there was no significant difference in fibrinogen level, including other parameters between CD and labor groups (Table 2), which were consistent with the study results conducted by Yamada et al. [23]and Karlsson et al. [29].

The baseline measures of Quantra had very close mean and median values, and, overall, seemed to follow a normal distribution, as evaluated by the Q-Q plot and the histograms. Currently, there are few studies that report the normal range in pregnant women for a device utilizing SEER [13, 24]. Our results showed that means of CT, CTH and CTR parameters, including values at the 2.5th percentile and 97.5th percentile, in pregnant blood were found to be in normal reference range of Quantra. While those values of CS and PCS were normal to high when compared to the reference range of Quantra in non-pregnant blood. The results of FCS in our study were higher than its reference range. However, Kodali et al. demonstrated the means of QPlus parameters in 13 parturients, which approximated to the means of QPlus parameters in our study [24]. Both FCS values and fibrinogen levels were well correlated in our study and were in concordance with hyperfibrinogenemia during pregnancy.

Previous studies have demonstrated a strong correlation between fibrinogen levels measured by the Clauss method and functional fibrinogen assessments obtained via viscoelastic testing, including Quantra (FCS, *r* = 0.59–0.72), ROTEM (FIBTEM, *r* = 0.76–0.84), and TEG (Functional Fibrinogen, *r* = 0.68–0.71) [11, 13, 14]. Consistent with these findings, our study demonstrated a high correlation between fibrinogen level and FCS (*r* = 0.67) as shown in Fig. 2A. This result is in agreement with previous studies in pregnant women, which reported correlations of *r* = 0.72 and *r* = 0.77 [13, 29]. Moreover, these comparable correlation coefficients were reported in other study conducted in cardiac and orthopedic patients [11, 18, 30]. The strong correlation between FCS and fibrinogen can make the use of this parameter beneficial to early recognition of low fibrinogen levels. Hypofibrinogenemia (less than 2 g/L) has been shown to be the earliest and best predictor for progression from moderate to severe obstetric hemorrhage with a 100% positive predictive value [5, 6]. Additionally, Naik et al. provided the FCS value < 1.9 hPa as fibrinogen < 2 g/L with a sensitivity of 86% and specificity of 77% in the cardiac and major orthopedic patient populations [11]. During severe obstetric hemorrhage, fibrinogen is rapidly consumed. Early detection of hypofibrinogenemia and replacement of fibrinogen with a target level of more than 2 g/L are emphasized in the management of postpartum hemorrhage [5]. This task can be achieved by using point-of-care testing when time is critical.

As previously described by Groves et al., the PCS parameter is calculated by subtracting FCS from CS. Although it has been demonstrated that PCS is independently associated with platelet count and platelet function, this parameter does not directly measure the platelet count [19]. Our study demonstrated a moderate correlation (Fig. 2B), between PCS and platelet count (*r* = 0.52). This finding was in agreement with the result from a previous report that included pregnant women [13] and cardiac surgery cohort which reported by Huffmyer et al. (*r* = 0.48) [30]. Although our study demonstrated a moderate correlation between PCS and platelets, this measurement could be used to discern if there is a need for sending additional laboratory tests, for example a complete blood count (CBC), for assessing platelets. Naik et al. demonstrated that the PCS < 12.1 hPa was associated with platelet < 80 × 10^9^/L with 100% negative predictive value and PCS < 11.2 hPa as platelet < 50 × 10^9^/L with a sensitivity of 100% and specificity of 87%, including a 100% negative predictive value in the cardiac and major orthopedic patient populations [11]. Thus, a PCS < 12.1 hPa may indicate the need for ordering a CBC, whereas a PCS < 11.2 hPa in the setting of severe hemorrhage may indicate the need for ordering platelets to be transfused.

The data presented in Fig. 2 C showed a weak correlation between CT and PTT (*r* = 0.38), which was in concordance with the result of the study conducted in patients who underwent spine surgeries (*r* = 0.34) [18]. Whereas Kodali et al. showed a moderate correlation between CT and PTT. This may be due to different numbers in sample size. They conducted the study on 13 pregnant women, while our study enrolled 170 pregnant women into the analysis [24]. Moreover, the weak correlation that we found can be related to the method of testing. Conventional laboratory (PT, PTT) uses plasma [31], whereas Quantra uses whole blood to assess clot time [15]. More studies may be needed to explore this issue.

The slow turnaround time of standard laboratory coagulation tests which can be up to 60 min has become an obstacle to early detection of hypofibrinogenemia and management of severe obstetric hemorrhage [9–11]. The POCVT has been designed to rapidly provide coagulation status. Quantra, a novel platform, was reported to provide results quicker than standard laboratory coagulation tests and other platforms of POCVT (TEG^®^5000, ROTEM^®^-Delta) [13, 32]. Most studies, including our study, reported time to result for Quantra of less than 13 min [18, 30, 32]. In this study, we received the results within 12 min, which was 36 (28,, 48) minutes faster than the standard laboratory coagulation tests. Therefore, it is suitable to be a near-patient coagulation monitoring device.

There are few limitations in our study. Parturients with platelet disorders, clotting factor abnormalities, and parturients who were on anticoagulants were not included. If we include these patient groups, the results from Quantra may have more variations in values; however, the correlation between Quantra parameters and standard laboratory coagulation tests may be higher. Additionally, we can determine the sensitivity and specificity of Quantra parameters if we have both normal pregnant women and pregnant women with coagulopathy. Further studies are needed to perform in bleeding cases as well to validate this novel ultrasound-based technology POCVT in the setting of severe obstetric hemorrhage. Moreover, the clinical implication of Quantra and platelet abnormalities are needed to be explored to guide the anesthesiologist not only in the setting of massive blood loss but also in the setting of performing regional anesthesia in pregnant women.

## Conclusions

Quantra is a useful point-of-care monitoring device that can rapidly provide a measure of the coagulation status. Due to a physiological hypercoagulable state during pregnancy, the baseline of Quantra parameters that we established had some changes from the normal reference range. Furthermore, there was a strong correlation between FCS and fibrinogen level which can aid clinicians in early recognition of hypofibrinogenemia during severe obstetric hemorrhage and can be used as an additional adjunct during replacemSSent of fibrinogen to reduce blood product use and improve maternal outcomes with fewer complications.

## Data Availability

No datasets were generated or analysed during the current study.

## Abbreviations

BMI: Body mass index
CBC: Complete blood count
CD: Cesarean delivery
CLSI: Clinical Laboratory Standard Institute
CS: Clot Stiffness
CT: Clot Time
CTH: Clot Time with Heparinase
CTR: Clot Time Ratio
FCS: Fibrinogen Contribution to clot Stiffness
IFCC: International Federation of Clinical Chemistry
INR: International normalized ratio
IQR: Interquartile range
PCS: Platelet Contribution to clot Stiffness
POCVT: Point-of-care viscoelastic testing
PPH: Postpartum hemorrhage
PT: Prothrombin time
PTT: Partial thromboplastin time
ROTEM: Rotational thromboelastometry
SD: Standard deviation
SEER: Sonic Estimation of Elasticity via Resonance
TEG: Thromboelastography
